# Compliance with the WCRF/AICR Recommendations in Qualitative Adaptation and the Occurrence of Breast Cancer: A Case-Control Study

**DOI:** 10.3390/cancers16020468

**Published:** 2024-01-22

**Authors:** Beata Stasiewicz, Lidia Wadolowska, Maciej Biernacki, Malgorzata Anna Slowinska, Ewa Stachowska

**Affiliations:** 1 Department of Human Nutrition, Faculty of Food Sciences, University of Warmia and Mazury in Olsztyn, Sloneczna 45f, 10-718 Olsztyn, Poland; lidia.wadolowska@uwm.edu.pl (L.W.); malgorzata.slowinska@uwm.edu.pl (M.A.S.); 2 Department of General and Minimally Invasive Surgery, University of Warmia and Mazury in Olsztyn, 10-045 Olsztyn, Poland; maciej.biernacki@uwm.edu.pl; 3 Department of Human Nutrition and Metabolomics, Pomeranian Medical University, 71-460 Szczecin, Poland

**Keywords:** breast cancer, prevention, WCRF/AICR, recommendations, diet, women

## Abstract

**Simple Summary:**

Studies regarding the current recommendations of the World Cancer Research Fund (WCRF) and the American Institute of Cancer Research (AICR) in the context of breast cancer in women are limited. The aim of this study was to evaluate the association between the WCRF/AICR score in the qualitative adaptation and the occurrence of breast cancer in peri- and postmenopausal women. The obtained results indicate the benefits of compliance with the WCRF/AICR recommendations in a qualitative adaptation by reducing the occurrence of breast cancer in peri- and postmenopausal women.

**Abstract:**

Purpose: The aim of the study was twofold: (1) the qualitative adaptation of the 2018 WCRF/AICR (QAd-WCRF/AICR) score, and (2) the assessment of the association between the level of compliance with the WCRF/AICR recommendations and the occurrence of breast cancer in peri- and postmenopausal women. Methods: This case–control study involved 420 women, aged 40–79 years, from northeastern Poland, including 190 newly diagnosed breast cancer cases. Data related to the WCRF/AICR recommendations were collected in face-to-face interviews with 409 women, including 179 women with breast cancer. The frequency of food consumption data were collected using the FFQ-6^®^ and KomPAN^®^ questionnaires. Body weight, height, and waist circumference were measured. The QAd-WCRF/AICR score (range: 0–8 points) was calculated on the basis of eight components, including two components from to the WCRF/AICR recommendations: (1) body mass index (BMI), and (2) waist circumference, with six components expressed qualitatively: (3) overall physical activity, as well as the frequency of the consumption of (4) vegetables/fruits/whole grains/nuts/seeds/legumes, (5) highly processed foods, including fast foods/sweets/instant soups, (6) red/processed meat, (7) sweetened/energy drinks, and (8) alcohol. Logistic regression analysis was performed to assess the occurrence of breast cancer. Results: The moderate (4–5 points) and maximal (6–8 points) compliance with the qualitative adaptation of the WRCF/AICR recommendations reduced the odds of breast cancer by 54% and 72%, respectively, compared to the results noted for minimal compliance (≤3 points). Lower odds of breast cancer were associated with moderate or high physical activity, consumption of a minimum of four serving per day of vegetables/fruits/whole grains/nuts/seeds/legumes, and limiting the consumption of highly processed food/fast foods and red/processed meat to a maximum of 1–3 times/month. Higher odds of breast cancer were associated with a higher waist circumference and alcohol abstinence. Conclusions: These findings may prove useful in establishing cancer prevention recommendations based on simple suggestions regarding the frequency of food consumption.

## 1. Introduction

Globally, breast cancer (BC) is the most common cancer incidence and cause of mortality in women [1]. According to the latest GLOBOCAN statistics, over 2 million new BC cases (24.5% of total female cancer cases) and about 685 thousand new BC deaths (15.5% of total female cancer deaths) were reported worldwide in 2020 [1]. On average, one in eight women worldwide will be diagnosed with breast cancer during her life. BC is the leading diagnosed cancer among women in every European country, including Poland, where over 18 thousand of these tumours were diagnosed (23.8% of total female cancers) in 2020. Regarding cancer death among Polish women, BC ranks second [2,3]. The occurrence of breast cancer increases with age. Approximately 80% of BC cases were diagnosed among peri- and postmenopausal women over 50 years of age [3,4,5,6]. Despite the continuous improvements in oncology, cancers constitute the leading major burden on public health both in medium and high-income countries, just after cardiovascular diseases [2]. Due to unfavourable data statistics and the constantly increasing incidence of cancer, it is necessary to take effective preventive precautions and strategies.

In the complex aetiology of BC, besides genetic defects, a number of modifiable lifestyle-related factors have been identified. The latter is estimated to be approx. 21% in premenopausal women and almost 35% in postmenopausal women [7]. There is growing evidence that changes in diet and physical activity could prevent 25% to 30% of breast cancer cases [7]. According to the WCRF/AIRC report, there is strong evidence (convincing) that adult weight gain and body fatness, mainly abdominal adiposity, increase postmenopausal breast cancer [5,6]. In BC prevention, a healthy body weight expressed by BMI should range between 18.5 and 24.9 kg/m^2^ [5,6]. Globally, approximately 39% of women are overweight (BMI ≥ 25 kg/m^2^) [2]. Hence, the increase in cancer incidence can be seen as a result of the increasing prevalence of obesity worldwide. Alcohol is the next strong factor that increases the risk of BC for both premenopausal (probable evidence) and postmenopausal (convincing evidence). women It was estimated that the daily consumption of 10 g of ethanol increases the risk of BC by up to 12% [5,6]. This is caused by the pro-oestrogenic effect of ethanol and the genotoxic effects of its metabolite—acetaldehyde [7,8,9].

Factors that have a protective effect against BC include physical activity. There is strong evidence (probable) that vigorous physical activity decreases premenopausal breast cancer, and moderate or vigorous physical activity decreases postmenopausal breast cancer [5,6]. Recommended physical activity for adults is at least 150 min/per week, and this also allows for healthy body weight maintenance [2]. The next factor that likely decreases the overall risk of breast cancer is breastfeeding [5,6]. Lactation is associated with prolonged amenorrhea and infertility, and thus reduced lifetime exposure to sex hormone levels, as high concentrations increase the risk of BC [5,6,7]. It is recommended that mothers breastfeed for at least six months [5,6]. Regarding dietary factors, including foods and nutrients related to BC, evidence is limited. Based on the number of studies that have suggested the protective anti-cancer role of a plant-based diet, it is recommended to increase the consumption of vegetables and fruits above 400 g/day, along with an increased consumption of whole grains and beans [5,6]. The cancer prevention recommendations also involve limiting the consumption of red meat to below 500 g per week and avoiding the consumption of highly processed foods with a high content of starches, fat, or sugars, including fast foods or sugar-sweetened drinks [5,6].

The development of cancer is determined by the number of accumulated environmental and lifestyle risk factors and their interactions with the internal human genetic, metabolic, and reproductive factors [7]. Therefore, in addition to assessing the exposure of individual known cancer risk factors, an estimate of their cumulative impact is needed. In this context, the World Cancer Research Fund (WCRF) and the American Institute of Cancer Research (AICR) developed in 2007 and actualised in 2018 the WCRF/AICR score, based on scientific evidence of modifiable lifestyle cancer risk factors [4,5,6]. The WCRF/AICR score comprehensively incorporates eight recommendations for cancer prevention related to diet, physical activity, and body weight management [5,6]. The dietary recommendations include limiting the consumption of red meat, high-processed foods and beverages, and alcohol and increasing the consumption of plant foods. One special recommendation concerned mothers and breastfeeding [5,6].

Recently published data reveals that adherence to the WCRF/AICR recommendations is associated with reduced breast cancer risk [10,11,12,13,14,15,16,17,18,19]. However, there is a high heterogeneity of available study results [20,21,22,23]. To the authors’ best knowledge, no studies have so far been published regarding the 2018 WCRF/AICR score among Polish women. Furthermore, it is sometimes difficult to assess compliance with recommendations expressed quantitatively, including physical activity and food consumption, to calculate the WCRF/AICR score. The qualitative adaptation of the WCRF/AICR score may be an alternative and very useful approach in the cancer prevention strategy. Considering the above, the aim of the study was twofold: (i) to develop the qualitative-adapted version of the 2018 WCRF/AICR (QAd-WCRF/AICR) score, and (ii) the assessment of the association between the level of adherence to the QAd-WCRF/AICR score, as well as of compliance with its specific recommendations, with the occurrence of breast cancer in peri- and postmenopausal women.

## 2. Materials and Methods

### 2.1. Cancer-Control Sample Collection

**Figure 1:**
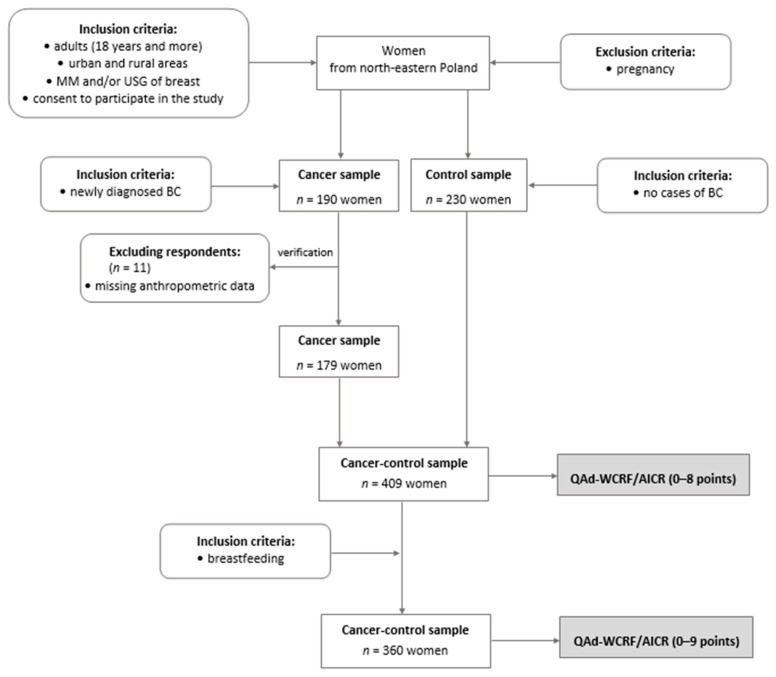
Flow chart of study design and sample collection. BC—breast cancer; MM—mammography; USG—ultrasonography; QAd-WCRF/AICR—the qualitative adaptation of the 2018 WCRF/AICR score; the stages of the study are shaded.

This study was a part of the case–control research conducted in 2014–2017 among 420 peri- and postmenopausal women from northeastern Poland. The initial control sample was matched with cases by age and BMI to reduce the variability of the basic input data in the study. All details regarding matching samples were provided elsewhere [24]. The complete set of data used to calculate the QAd-WCRF/AICR (0–8 points) was collected for 409 women, aged 40.0–79.9 (mean 60.0) years, including 179 breast cancer (BC) cases (cancer sample) and 230 women without breast cancer or any breast pathology (control sample; Figure 1). An additional inclusion criterion necessary to calculate the QAd-WCRF/AICR, expressed in the range of 0–9 points, was breastfeeding, which was exhibited by 360 women, including 167 BC cases and 193 controls.

All breast cancer cases were diagnosed and histologically confirmed at the surgical oncology ward of the Warmia-Masuria Cancer Centre of the Ministry of the Interior and Administration Hospital, Olsztyn, Poland. BC cases were included in the study within a month after primary diagnosis, before any treatment or surgical intervention. The most frequently diagnosed cases of breast cancers were luminal A subtype tumours, with positive oestrogen (ER+) and progesterone receptor status (PR+) and negative human epidermal growth factor receptor 2 (71.2%). The control sample was recruited based on the mammography (MM) and/or breast ultrasonography (USG) national screening program performed up to six months before recruitment in the study. 

### 2.2. The Qualitative-Adapted 2018 WCRF/AICR (QAd-WCRF/AICR) Score

The original 2018 WCRF/AICR score [25,26] was modified for the present analysis as a qualitative-adapted WCRF/AICR (QAd-WCRF/AICR) score (Table S1). The proposed QAd-WCRF/AICR score was developed using eight components, including two components from to the WCRF/AICR recommendations: (1) body mass index (BMI), and (2) waist circumference. Body weight and height, as well as waist circumference, were measured. In developing the QAd-WCRF/AICR score, the next six components were expressed qualitatively instead of quantitatively in comparison with the original score: (3) physical activity, determined on the basis of physical activity during work and leisure time (Table S2) [27,28], and the frequency of consumption of (4) vegetables/fruits/whole grains/nuts/seeds/legumes, as well as the consumption of (5) highly processed food, including fast foods/sweets/instant soups, (6) red/processed meat, (7) sweetened/energy drinks, and (8) alcohol. Data regarding physical activity were obtained using the Dietary Habits and Nutrition Beliefs Questionnaire developed by the Committee of Human Nutrition, Polish Academy of Sciences (KomPAN^®^) [29]. Dietary data were assessed using a validated 62-item Food Frequency Questionnaire (62-itemFFQ-6^®^) and KomPAN^®^ [30]. Respondents reported their usual food consumption frequency during the previous year, before participation in the study. To reduce the likelihood of reverse causation, all respondents involved in this study declared that they had made no changes in lifestyle or dietary habits in recent years. All face-to-face interviews were conducted using show cards with a list of six categories of food frequency consumption to choose from. For some foods, e.g., fruits and vegetables, besides questions about the frequency of consumption of single food items, there were also questions regarding the total consumption of these food groups, which made it possible to verify the reliability of the answers provided. The frequency consumption was recalculated and expressed as times/day as follows: ‘never or almost never’ = 0; ‘once a month or less’ = 0.025; ‘several times a month’ = 0.1; ‘several times a week’ = 0.571; ‘daily’ = 1; ‘several times a day’ = 2 times/day [30]. Some of the food items were combined by summing their frequency consumption into food groups and again expressed in food frequency categories, as shown in Supplementary Table S1. The proposed categories of food frequency consumption corresponded to the original categories, expressed quantitatively [25].

Depending on the level of compliance with the recommendations, points were assigned to categories in the individual score components, according to the criteria provided by Shams-White et al. [25] (Table S1). Compared to the original score, in the qualitatively adapted version, the scoring system employed each recommendation as a single item, and none of the recommendations comprised two subitems. For full, partial, and lack of compliance with the WCRF/AICR recommendations, 1, 0.5, and 0 points were assigned, respectively. The QAd-WCRF/AICR score was calculated as the sum of points of each recommendation and expressed in a range from 0 to 8 points. Optionally, the QAd-WCRF/AICR score also assessed the longest breastfeeding period for mothers (never, ≤6 months, >6 months) as a special recommendation, expressed in the range from 0 to 9 points. The higher values of both scores indicated higher compliance with the cancer prevention recommendations. For further analyses, in order to enable the comparison of the results with those from other studies, the overall scores were categorised. The categories of the QAd-WCRF/AICR score (0–8 points) were created using three levels: minimal (≤3 points), moderate (4–5 points), and maximal adherence (6–8 points), and two levels: lower (0–4 points) and higher adherence (5–8 points). The categories of the QAd-WCRF/AICR score (0–9 points) were also created using three levels: minimal (≤4 points), moderate (5–6 points), and maximal adherence (7–9 points), and two levels: lower (0–5 points) and higher adherence (6–9 points). Characteristics of the adherence to the QAd-WCRF/AICR Score (0–8 points) by its single recommendations are shown in Supplementary Table S3.

### 2.3. Statistical Analysis

The continuous variables, including the QAd-WCRF/AICR score (points), BMI (kg/m^2^), waist circumference (cm), and frequency of consumption of specific food items (times/day), were shown as means and standard deviations (SDs). For these variables, the differences between breast cancer cases and controls were verified using a Kruskal–Wallis test [31]. The QAd-WCRF/AICR score and its single components were also categorised and presented in sample percentages. The percentage distributions of the categorical variables were compared between groups using the Pearson Chi^2^ test, with Yates’s correction, as necessary [31].

Logistic regression analysis was performed to assess the odds ratio (OR) and 95% confidence interval (95% CI) of breast cancer occurrence in association with the adherence to the levels of the QAd-WCRF/AICR recommendations, as well as to estimate the OR of breast cancer in association with the compliance with single QAd-WCRF/AICR recommendations. The reference categories (OR = 1.00) were the control sample and the minimal or lower level of adherence to the QAd-WCRF/AICR recommendations or the lack of compliance with the QAd-WCRF/AICR single recommendations. The ORs of BC for a one-point increase in the QAd-WCRF/AICR score and one unit of QAd-WCRF/AICR single recommendations were also calculated. In assessing the breast cancer occurrence by level of adherence to the QAd-WCRF/AICR recommendations, two models were created: the crude model (OR_crude_), and the model adjusted for the set of potential confounders (OR_adj_). The list of the literature-based selected potential confounders [5,6] was provided in the logistic regression analysis of the Results section. In the ORs of BC occurrence assessment by the compliance with single QAd-WCRF/AICR recommendations, an additional multi-variable adjusted model was created (OR_m-adj_), including the set of confounders of the OR_adj_ model and the remaining components of the QAd-WCRF/AICR score. The level of significance of OR was verified using the Wald test [31]. Statistical analyses were performed using the STATISTICA software (version 13.0 PL; StatSoft Inc., Tulsa, OK, USA; StatSoft, Krakow, Poland). The level of statistical significance was defined at a *p*-value < 0.05.

## 3. Results

The baseline sample characteristics regarding the adherence to the QAd-WCRF/AICR recommendations (0–8 points) are shown in Table 1. Women with maximal compliance with the WCRF/AICR recommendations were less likely to have breast cancer; exhibited a lower BMI, waist circumference, waist-to-heigh ratio, and fat mass; were more physically active, including participation in physical activity at work and in their leisure time; had a higher socioeconomic status, including a higher education level; more often came from cities with more than 100,000 inhabitants; and were more likely to be nulliparous. Most women were postmenopausal (85.6%) and experienced an average economic situation (70.4%). About half of the participants experienced chronic disorders (57.0%) and were current or former smokers (51.8%). Only 4.2% of women abused alcohol (Table 1).

### 3.1. QAd-WCRF/AICR Recommendations among Breast Cancer Cases and Controls

The comparative characteristics of the cancer and control (non-cancer) samples, by the adherence to the QAd-WCRF/AICR recommendations and its single compliances, are shown in Table 2. Compared to the controls, the breast cancer cases exhibited, on average, lower adherence to the QAd-WCRF/AICR recommendations, expressed in both the range from 0 to 8 points (3.8 vs. 4.3) and in the range from 0 to 9 points (4.5 vs. 5.0). The maximal adherence to these QAd-WCRF/AICR recommendations was obtained by 17.4% and 12.4% of controls and only 8.9% and 7.8% of BC cases, respectively. The average waist circumference was higher among BC cases than among controls (94.0 vs. 90.4 cm). More cases of breast cancer in comparison with controls had low overall physical activity (67.9 vs. 40.4%), consumed at least once per day red and processed meat (70.5 vs. 62.2%), and highly processed food, including fast foods/sweets/instant soups (26.8 vs. 16.1%), and declared alcohol abstinence (52.6 vs. 30.4%). There were fewer individuals with breast cancer than the controls who consumed vegetables/fruits/whole grains/nuts/seeds/legumes at least four times per day (27.4 vs. 38.3%). Individuals with cancer and the controls did not differ in BMI, frequency of consumption of sugar-sweetened and energy drinks, or the time of breastfeeding (Table 2).

### 3.2. QAd-WCRF/AICR Score and Breast Cancer Occurrence

The odds of BC occurrence were lower by 54% (OR: 0.46; 95% CI: 0.28–0.76; *p* = 0.0024; adjusted model), and 72% (OR: 0.28; 95% CI: 0.13–0.63; *p* = 0.0018; adjusted model) among women reporting moderate (4–5 points) and maximal (6–8 points) adherence to the QAd-WCRF/AICR recommendations (0–8 points), respectively, when compared to those reporting minimal adherence (≤3 points), as a reference (Table 3). Regarding the two-level division of the adherence to the QAd-WCRF/AICR recommendations, the odds of BC occurrence were lower by 51% (OR: 0.49; 95% CI: 0.31–0.76; *p* = 0.0015; adjusted model) among women with a higher adherence (5–8 points) to the QAd-WCRF/AICR recommendations (0–8 points), when compared to those with a lower adherence (0–4 points) as a reference. A one-point increase in the QAd-WCRF/AICR Score decreased the odds of BC occurrence by 31% (OR: 0.69; 95% CI: 0.56–0.86; *p* = 0.0007; adjusted model). In regards to the moderate (5–6 points) and maximal adherence (7–9 points) to the QAd-WCRF/AICR recommendations expressed in the range of 0–9 points, the odds of BC occurrence were lower by 65% (OR: 0.35; 95% CI: 0.13–0.98; *p* = 0.0445; adjusted model) and 83% (OR: 0.17; 95% CI: 0.06–0.54; *p* = 0.0023; adjusted model), respectively, when compared to those with minimal adherence (≤4 points) as a reference. Regarding the two-level division of the adherence to the QAd-WCRF/AICR recommendations, the odds of BC occurrence were lower by 55% (OR: 0.45; 95% CI: 0.27–0.75; *p* = 0.0020; adjusted model) among women with a higher adherence (6–9 points) to the QAd-WCRF/AICR recommendations (0–9 points), when compared to those with lower adherence (0–5 points) as a reference. A one-point increase in the QAd-WCRF/AICR Score (0–9 points) decreased the odds of BC occurrence by 29% (OR: 0.71; 95% CI: 0.57–0.88; *p* = 0.0019; adjusted model). All of these associations support the results from the crude models (Table 3).

### 3.3. QAd-WCRF/AICR Single Recommendations and Breast Cancer Occurrence

The results regarding breast cancer occurrence after compliance with the single components of the QAd-WCRF/AICR recommendations are shown in Table 4. The lower odds of BC occurrence were associated with moderate or high overall physical activity (OR: 0.33; 95% CI: 0.20–0.56; *p* < 0.0001; multi-variable adjusted model; reference: low physical activity), increased consumption of vegetables/fruits/whole grains/nuts/seeds/legumes more than four times/day (OR: 0.36; 95% CI: 0.15–0.86; *p* = 0.0208; multi-variable adjusted model; reference: <2 times/day), and restricted consumption of highly processed food including fast foods/sweets/instant soups (OR: 0.36; 95% CI: 0.15–0.83; *p* = 0.0165; multi-variable adjusted model; reference: ≥1 time/day), and red and processed meat (OR: 0.48; 95% CI: 0.25–0.91; *p* = 0.0235; adjusted model; reference: ≥1 time/day) to 1–3 times/month or less The higher odds of BC occurrence were associated with a one-point increase in waist circumference (OR: 1.04; 95% CI: 1.01–1.07; *p* = 0.0206; multi-variable adjusted model), a one-point increase in the frequency of consumption of red and processed meat (OR: 1.31; 95% CI: 1.01–1.71; *p* = 0.0449; adjusted model), and with the alcohol abstinence (OR: 2.98; 95% CI: 1.78–4.98; *p* < 0.0001; multi-variable adjusted model).

## 4. Discussion

To the authors’ best knowledge, this was the first study to evaluate the associations of compliance with the qualitative-adapted version (QAd-WCRF/AICR) of the 2018 WCRF/AICR recommendations with breast cancer occurrence. The findings from the present study confirm the known benefits of following most of the WCRF/AICR recommendations, including being physically active and having a normal waist circumference, in reducing breast cancer occurrence in peri- and postmenopausal women. The results also provide meaningful insights for future cancer prevention strategies in establishing recommendations based on qualitative data regarding food frequency consumption. These recommendations involve the consumption of vegetables/fruits/whole grains/nuts/seeds/legumes at least four times per day and limiting the consumption of highly processed foods, including fast foods/sweets/instant soups, and red and processed meat to a maximum of several times a month.

### 4.1. QAd-WCRF/AICR Score and Breast Cancer

The average adherence to the WCRF/AICR recommendations was significantly lower among BC cases than among controls, which supports the findings from a previous Polish study based on the recommendations from 2007 [32]. In the current study, the odds of BC occurrence were lower by 72% among women who followed at least six suggestions in the QAd-WCRF/AICR recommendations (0–8 points), compared to those who met three or fewer. All available studies dealing with this issue have evaluated the association between compliance with the WCRF/AICR recommendations and breast cancer risk, based on quantitative data [10,11,12,13,14,15,16,17,18,19,20,21,22,23]. Despite these studies, results similar to those from the present were obtained by Barrios-Rodriguez in a prospective cohort of Spanish women, in which the highest compared to the lowest level of adherence (>5 vs. ≤3 points) to the 2018 WCRF/AICR recommendations (0–7 points) was associated with a 73% lower risk of postmenopausal breast cancer [10]. This significant inverse association was also observed in a number of previous studies, including case–control studies [13,17,19], prospective cohort studies [11,12,14,15,16,33], and recent meta-analyses [18,19], in which, for the highest vs. the lowest levels of adherence to the WCRF/AICR score category, the risk of BC was from 20% to 60% lower. The relatively wide range of values for these ratios is probably due to differences in the construction of the WCRF/AICR score, including the number of 2018 WCRF/AICR recommendations or previous 2007 recommendations taken into account. Further, there were differences in the operationalizing of single recommendations, which were assigned different values on a scale from 0 to 1 point, depending on the degree of compliance with the recommendation, including partial points, e.g., 0, 0.25 or 0.5, in some studies [10,11,12,13,14,15,16,17,18,19,20,21,22,23]. In the previously mentioned studies, different approaches were used to determine cut-off points in regards to the WCRF/AICR score and to define the a priori low and high categories, based on the literature review, or on the calculated a posteriori tertiles. In addition, apart from differences in design, these studies involved various age groups of women, including premenopausal or postmenopausal, or the risk of breast cancer was calculated overall [10,11,12,13,14,15,16,17,18,19,20,21,22,23]. In contrast to the results of the current study, a significant association between adherence to the WCRF/AICR recommendations evaluated categorically and breast cancer risk was not observed in the European Prospective Investigation into Cancer and Nutrition (EPIC) cohort study, the Swiss National Nutrition Survey, the Canadian National Breast Screening Study, or the Black Women’s Health Study [20,21,22,23]. In these studies, the WCRF/AICR score reduced BC only in the continuous model, and these associations were weaker than those in the own study.

### 4.2. QAd-WCRF/AICR Single Recommendations and Breast Cancer

The strong inverse association of adherence to the QAd-WCRF/AICR recommendations with breast cancer occurrence resulted from compliance with the single WCRF/AICR recommendations. Analyses involving individual QAd-WCRF/AICR components have shown that meeting most of the single recommendations related to diet and lifestyle significantly contributed to the reduction of BC occurrence. These recommendations involve increasing the consumption of plant-based foods and limiting the consumption of highly processed foods, as well as red and processed meat. Next, the beneficial importance of physical activity was emphasized. In turn, the factors that increased breast cancer occurrence were an increase in waist circumference and alcohol abstinence. The obtained findings, excluding alcohol abstinence, support results from previous studies regarding WCFR/AICR recommendations based on quantitative data.

#### 4.2.1. Plant-Based Foods and Breast Cancer

The consumption of plant-based foods, including fruits, vegetables, whole grains, nuts, seeds, and legumes, at least four times per day was associated with a 64% decrease in BC occurrence, when compared with results derived from this consumption two times per day or less. This frequency of consumption category is the qualitative equivalent of the 2018 WCRF/AICR recommendation for eating a diet rich in whole grains, vegetables, fruit, and beans, which means an intake of at least 400 g per day of fruits and vegetables and at least 30 g per day of total dietary fibre [5,6]. The results from the present study are consistent with results from other case–control studies from Spain [17], Italy and Switzerland [19], and South Africa, where meeting this recommendation resulted in a 34%, 37%, and 45% decreased risk of breast cancer, respectively [13]. The inverse association between plant-based food consumption and breast cancer risk could be explained by several potential mechanisms. These foods are rich sources of fibre, which may prevent breast cancer by binding and excreting oestrogens, decreasing their circulating levels, and helping maintain a normal body weight [34]. Plant-based foods also contain vitamins, minerals, and bioactive phytochemicals, including flavonoids—natural antioxidants that reduce the concentration of free radicals in the blood, reducing oxidative stress and inflammation [35]. This protective effect could also result from the beneficial effect of high-fibre foods on the microbiome composition [36].

Korn et al. [14] found that a plant-based diet was associated with a reduced risk of cancer only in the never or current smokers and not among former smokers. However, there were three types of cancers considered in this study: breast, lung, and colorectal. Hence, the contribution of smoking as a proven risk factor for lung cancer had a significant impact on the obtained results [14]. Catsburg et al. [20] reported that a significant decrease in the risk of breast cancer resulting from the consumption of plant-based foods only occurred for whole grain, and not refined grain, consumption. Whole grain foods, unlike refined foods, stabilise glycaemic and insulin levels, thus preventing the increase in the concentration of insulin-like growth factor-1 IGF-1, a risk factor for breast cancer [35]. In contrast to the present results, a significant impact of plant-based food consumption on the reduction of breast cancer risk was not observed in all studies [10,11,12,15,21,22]. Based on this, according to the WCRF/AICR report [6], there is limited evidence that plant-based foods decrease the risk of breast cancer. This may indicate that achieving the healthy characteristics of the diet is not sufficient to reduce cancer risk, suggesting the need to evaluate the diet as a whole, including the food intake that should be limited. Moreover, in the complex aetiology of breast cancer, there are interactions between food molecules that are difficult to evaluate [9].

#### 4.2.2. Highly Processed Foods and Breast Cancer

##### Soft and Energy Drinks and Breast Cancer

Limiting the consumption of highly processed foods, including fast foods, sweets, and instant soups, to 1–3 times per month or less decreased BC occurrence by 64% compared to results for those consuming these items at least one or more times per day. Similarly, Turati et al. [19] reported that limiting the consumption of fast foods and other ultra-processed foods high in fat starches or sugars decreases the risk of BC by 25% (limiting energy density to 125 kcal/100 g/day or less vs. 175 kcal/100 g/day or more). Highly processed foods include high energy-dense foods, which are defined as foods containing 225 kcal or more per 100 g [4]. This group includes fast foods, sweets, and salty snacks with high sugar and/or fat content. Therefore, these foods might have an effect on BC risk through promoting weight gain. However, in some studies, there were no significant associations between fast food and other ultra-processed and high-energy-dense food consumption with BC risk [10,11,12,13,14,15,21]. Thus, the evidence of an association between the consumption of processed food and BC is still weak [6]. A significant and positive association between the consumption of high-density foods and BC was observed by Castello et al. [37], but only in premenopausal women. These findings indicate the increased consumption of this type of food in young women due to the higher adherence to a Western-style diet.

One of the food items included in the group of highly processed foods according to the previous 2007 WCRF/AICR recommendations, and then labelled as a separate recommendation in the 2018 WRCF/AICR, were sugary soft drinks. Turati et al. [19] showed that limiting the consumption of sugar-sweetened drinks reduced the risk of breast cancer by 26% (≤250 vs. >250 g/day) and 32% (0 vs. >250 g/day), respectively. A further reduction in the risk of breast cancer by 58% was possible by avoiding sugary drinks and additionally limiting the consumption of energy-dense foods ≤125 kcal/100 g (vs. 175 kcal/100 g/day or more and >250 g/day of sugary drinks) [17]. In contrast to the studies mentioned above, the present study did not find any association of breast cancer occurrence with the consumption of sugar-sweetened and energy drinks. This may be due to the very low consumption of these foods by Polish women over 50 [33]. In this study, 94% of women consumed these beverages only several times a month or less. This could also be the reason for the lack of a significant association between the consumption of sugary drinks and breast cancer in most available studies [10,13,14,15,21,22].

#### 4.2.3. Red Meat and Breast Cancer

In the current study, limiting the consumption of red and processed meat to 1–3 times per month or less decreased BC occurrence by 52% compared to the results for consuming these items at least one or more times per day. Some studies found a weaker, but still significant, association when limiting red meat intake to <500 g/week, which reduced breast cancer risk by 18–21%, according to the WCRF/AICR [11,20,22]. Red meat is a rich source of heme iron, so its frequent consumption may have an unfavourable pro-oxidant effect on cells. The consumption of fried and grilled red meat should be avoided, especially due to the content of heterocyclic amines and polycyclic aromatic hydrocarbons that may contribute to the process of carcinogenesis [4,5]. However, these hypotheses require confirmation, and positive associations with breast cancer are often the result of meat consumption as a marker of an overall unhealthy lifestyle.

In present analyses, limiting red meat consumption did not result in a significant reduction in the incidence of breast cancer, after adjustment for the remaining WCRF/AICR recommendations. It seems that following other recommendations, such as consuming plant-based foods more frequently, could potentially help reduce the risk of breast cancer. [9]. Moreover, many studies failed to show significant associations between red meat consumption and breast cancer [10,12,13,15,17,19,21,37]. Hence, there is limited, non-conclusive evidence that the consumption of red and processed meat is a risk factor for breast cancer [6].

#### 4.2.4. Alcohol and Breast Cancer

Concerning diet-related factors and breast cancer, strong evidence was obtained only for the negative impact of alcohol consumption [6]. Currently, there is no established dose of alcohol intake that is safe for health. Some epidemiological studies reported that the risk of breast cancer was reduced by 10% by limiting alcohol consumption to no more than one alcoholic drink per day [20], and by 26% for non-drinkers [19] versus the risk for those consuming more than one drink per day. The carcinogenic effect of alcohol is mainly caused by toxic acetaldehyde, a product of alcohol metabolism that reaches different target tissues. The current evidence indicates that regular alcohol consumption may reduce the absorption of nutrients, including folic acid, and increase the concentration of sex hormones, mainly oestrogens. Moreover, alcohol may increase the permeability of cell membranes to other toxic substances, increase the concentration of reactive free oxygen radicals, and cause epigenetic disorders, including methylation changes in deoxyribonucleic acid (DNA) [35].

Surprisingly, the current results are contrary to these findings. Women who declared alcohol abstinence had almost three times greater BC occurrence than women who drank alcohol occasionally, less than one time per day. This result could be partially explained by the study design and method used in the dietary data collection. These data were collected from the 12 months before the participants were enrolled in the study. Women who declared abstinence, despite no changes in food consumption over recent years, may have consumed alcohol more often earlier. Cancer is the result of the influence of a number of factors over many years [7,8]. Further, women who abuse alcohol often declare abstinence due to shame, or women who abused alcohol many years ago could become teetotallers. Moreover, the average alcohol consumption among Polish postmenopausal women was relatively low and was reported to occur several times a month. None of the women among the controls consumed alcohol one time per day or more frequently. Therefore, the difference between the studied and reference frequency categories was insufficient to obtain a positive association between alcohol consumption and breast cancer occurrence. Thus, the current results do not suggest any benefits from alcohol consumption. For similar reasons, many studies did not show a significant association between alcohol consumption and the risk of breast cancer [10,11,13,15,17,21,22,37].

#### 4.2.5. Body Weight Status and Breast Cancer

Overweight and obesity are the results of a chronic positive energy balance through excessive energy intake and inadequate energy expenditure. There is strong, convincing evidence that body fatness and adult weight gain increase the risk of postmenopausal breast cancer [6]. In most of the available studies, the WCRF/AICR recommendation to ‘be a healthy weight’ was expressed in BMI. However, similar to the current results, many other studies did not find significant associations between BMI and breast cancer risk [10,11,13,19,20,21,22,37]. There were no significant differences between BC cases and controls in regards to BMI. The average BMI value among Polish women was 27.9 kg/m^2^, which indicates overweight. This confirms that BMI is not a sufficient indicator in assessing body weight status. This parameter does not indicate body fatness. In contrast to the present results, it was shown in several studies that maintaining body weight in the normal range of BMI, between 18.5 and 24.9 kg/m^2^, compared to obesity (BMI > 30 kg/m^2^), was significantly associated with a decrease in postmenopausal BC by from 10% [15] and 15% [12] to 47% [17].

Women with abdominal obesity are at a particularly increased risk of breast cancer [38,39]. The abdominal adiposity could be assessed by waist circumference (WC). In the present study, a one-point increase in WC resulted in an increase in BC occurrence by 4%. Women with diagnosed breast cancer had a significantly higher average WC than did the controls (94.0 vs. 90.4 cm). In both groups, this parameter was greater than the maximum recommended 88 cm, which indicates a higher risk of metabolic disorders associated with cancer [38]. An inverse association between WC and breast cancer was also observed by Lee et al. [15] among Korean women, where a WC < 80 cm compared to ≥80 cm reduces the risk of BC by 12%. There are several mechanisms that link abdominal obesity and breast cancer in postmenopausal women. In middle-aged women, more adipose tissue is accumulated viscerally, where androgens are converted to oestrogens that induce the proliferation and inhibit the apoptosis of tumour cells [39]. Abdominal obesity is associated with the production of pro-inflammatory adipokines in fat tissues, which promotes insulin resistance and hyperinsulinemia. Insulin inhibits the synthesis of sex hormone-binding globulin, which leads to elevated levels of free oestradiol. Thus, these associations were more evident for hormone-dependent breast cancer [11].

#### 4.2.6. Physical Activity and Breast Cancer

According to the WCRF/AICR report, there is probable evidence that physical activity involving recreational, occupational, and household activities decreases the risk of postmenopausal breast cancer [6]. This is confirmed by the results of this study, in which moderate to high overall physical activity reduced BC occurrence by 67% compared to the results for low physical activity. Similarly, previous studies have shown that total moderate or vigorous physical activity was associated with a 17% to 40% lower risk of breast cancer [13,19,22]. To prevent cancer, the World Health Organization recommends that adults engage in at least 150 min of moderate to intense physical activity per week, or 75 min of vigorous physical activity, or a combination of both [5,6].

Physical activity through increasing overall energy expenditure and normal weight maintenance could reduce the risk of overweight/obesity, which is an important risk factor for postmenopausal breast cancer [39]. Besides the obesity-related biological pathways, physical activity also improved immune function and glucose tolerance, reduced insulin resistance, and increased the level of endogenous oestrogens [40,41,42]. However, not all studies observed a statistically significant association between physical activity and the risk of breast cancer [10,11,12,15,17,20,21,37]. These heterogeneity results may be due to some methodological differences, including methods of physical activity measurement and cut-off points regarding the definition of moderate or vigorous physical activity.

#### 4.2.7. Breastfeeding and Breast Cancer

According to the WCRF/AICR report [6], lactation probably decreases the risk of breast cancer overall. Due to amenorrhea and infertility, lactation reduces exposure to sex hormones, including androgens, which increase the risk of breast cancer. Moreover, the exfoliation of breast tissue and epithelial apoptosis can reduce breast cancer by removing cells with DNA damage. Regarding the menopausal status, the evidence of the protective effects of breastfeeding with regards to breast cancer was less conclusive [6].

In the present study, 360 women (88% of the total sample) declared that they had breastfed their infants. A breastfeeding span of longer than six months was declared by 47.8% of the women in the study. However, a significant association was not found between the duration of breastfeeding and breast cancer occurrence. Due to gaps in the respondents’ memory, an exclusive breastfeeding time was not included, which could affect the results obtained. The special recommendation, ‘breastfeeding your baby, if you can’, was taken into account in only a few studies and, similar to the results in the current study, no significant association was observed with breastfeeding and the incidence of breast cancer [13,15,19,37].

### 4.3. Strengths and Limitations

This study is the first in Central Europe to provide important insight into the role of the WCRF/AICR recommendations in breast cancer prevention. The authors proposed a qualitative modification of the original 2018 WCRF/AICR recommendations and created a qualitative-adapted WCRF/AICR (QAd-WCRF/AICR). This innovative approach allows for the extension of the use of this score in the absence of quantitative data. Qualitative data is easier to collect when respondents have some difficulty in determining portion sizes [43,44,45]. Determining portion sizes is time-consuming and often difficult due to respondents’ health or memory issues. The current analyses have not been limited to the assessment of the association between the QAd-WCRF/AICR score and BC occurrence. The authors also examined the link between BC occurrence and eight single WCRF/AICR cancer prevention recommendations, expressed qualitatively, as well as the special recommendation related to breastfeeding. Other strengths include the inclusion of a dietary and lifestyle assessment using the validated FFQs. All data were collected in face-to-face interviews, and the anthropometric measurements were performed, not declared. This increased the reliability of the data obtained, as well as the strength of the results [44,45]. Finally, in this study, a multi-variable adjusted model was created, including a set of many known and potential confounders related to the association between diet and lifestyle and breast cancer. The high agreement between the results from the crude and adjusted models suggests little interference by potential confounders [31]. Nevertheless, it would be beneficial to investigate potential confounders more thoroughly, especially in order to specify the number of cigarettes smoked per day among smokers, the types of chronic diseases experienced, e.g., diabetes, as well as the kind and dose of vitamin/mineral supplements and hormone-replacement therapy used. However, many respondents were unable to recall such data more precisely. In addition, all possible variables, including genetic factors or environmental pollution, that are difficult to measure, but which might have influenced the observed associations, were not taken into account. Thus, the possibility of residual confounding by unaccounted variables cannot be ruled out [31,44].

This study also has several limitations that should be considered. There are some internal limitations related to the case–control design of this study. First of all, the retrospective design of the study might introduce biases and challenges in recalling accurate data. Thus, the potential impact of selective memory and reporting bias on the obtained findings should be considered [43,44,45]. The self-reported food consumption is biased and may result in the over-reporting of healthy foods (e.g., fruits and vegetables) and the under-reporting of unhealthy foods (highly-processed foods with high fat and sugar content) [44,45]. However, this bias affects not only the FFQs, but also most dietary assessment methods, like 24-h recall, based on self-reported data [45]. To reduce the impact of memory and reporting bias on the obtained findings, the FFQs contained a list of foods typical in the diet of Poles, with adequate-to-high internal repeatability [30,44]. Moreover, to make it easier for respondents to answer, the interviews were conducted using show cards with a list of possible answers to choose from. Furthermore, the verification questions were used to identify both reliable and unreliable respondents and then to select the final dataset [28]. Second, the findings from the present study only highlight associations between compliance with dietary and lifestyle recommendations and cancer occurrence, but do not provide evidence of their role in cancer aetiology. The case–control design does not clarify the cause-effect association [44,45]. The next limitation of this study is that all data were obtained retrospectively, at 12e months before the diagnosis, while the carcinogenic process may have taken many years [42,44]. Due to gaps in the respondents’ memories, data could not be collected for an extended time period. To reduce the likelihood of reverse causation, all respondents involved in this study declared that they had made no changes in lifestyle and dietary habits in recent years. Moreover, only newly diagnosed breast cancer cases were included in the study. Another limitation of the study is the lack of a quantification of the portion sizes of food consumed among the breast cancer cases and the controls. Dietary data were collected using a food frequency questionnaire. FFQs are less precise compared to multi-day food records [44,45]. Although this method is not free from measurement error, previous studies confirmed the utility of FFQs in evaluating dietary behaviours, as well as the associations between them and chronic disease [24,43,44,45]. Lastly, the sample size was sufficient to achieve the study aim, but not large enough to generalize the obtained results. A relatively limited sample size might affect the results by overestimation or underestimation [44]. Moreover, a non-random sample selection may reduce the strength and quality of the evidence. The matching cases and controls often result in stronger diet–disease associations than those existing in real life [45]. A limited sample did not allow us to stratify the analyses according to the molecular subtype of breast cancer [46]. Nevertheless, the results were adjusted for the breast cancer hormone receptor status.

## 5. Conclusions

The results confirm the benefits of compliance with the WCRF/AICR recommendations, including being physically active and maintaining a normal waist circumference, in reducing the occurrence of breast cancer in peri- and postmenopausal women. The present study provides an interesting new discovery that the consumption of plant-based foods at least four times per day and limiting the consumption of highly processed foods and red meat to a maximum of several times a month is protective against breast cancer. These findings may prove useful in establishing cancer prevention recommendations based on simple messages regarding the frequency of food consumption. Although alcohol abstinence increased the BC occurrence, alcohol could not be considered beneficial in regards to the development of breast cancer. Following all of these recommendations provides greater benefits than the adherence to any one single recommendation due to their combined synergistic effects.

Nevertheless, more research, especially prospective cohort studies in large samples, is needed to support our findings regarding implementing the QAd-WCRF/AICR recommendations for cancer prevention across all age ranges and in different populations of women. The stratification for the specific breast cancer characteristics should be considered in further analyses, including the consideration of molecular cancer subgroups.

## Figures and Tables

**Table 1:** Sample characteristics regarding the adherence to the QAd-WCRF/AICR Score (% or mean (SD).

Variable	Total Sample	Adherence to the QAd-WCRF/AICR Score (0–8 points)			*p*-Value
		Minimal (≤3)	Moderate (4–5)	Maximal (6–8)	
Sample Size	409	109	244	56	
Breast cancer cases, %	43.8	61.5	39.3	28.6	<0.0001
Age (years), mean (SD)	60.0 (8.6)	61.4 (9.0)	59.6 (8.6)	59.0 (7.3)	0.1216
Age (years), %					
40.0–49.9	15.4	11.9	16.4	17.9	
50.0–59.9	29.8	28.4	31.6	25.0	0.4367
60.0–69.9	42.5	43.1	40.6	50.0	
70.0–79.9	12.2	16.5	11.5	7.1	
Menopausal status, %					
perimenopausal	14.4	12.8	15.6	12.5	
postmenopausal	85.6	87.2	84.4	87.5	0.7227
BMI ^a^ (kg/m^2^), mean (SD)	27.9 (5.0)	31.6 (4.6)	27.4 (4.5)	23.1 (2.1)	<0.0001
BMI ^a^ (kg/m^2^), %					
<18.5 (underweight)	0.7	0.9	0.4	1.8	
18.5–24.9 (normal weight)	29.6	1.8	29.9	82.1	
25.0–29.9 (overweight)	38.4	33.9	45.5	16.1	<0.0001
≥30.0 (obese)	31.3	63.3	24.2	0.0	
Waist circumference (cm), mean (SD)	92.0 (13.2)	102.6 (11.5)	90.3 (11.2)	78.4 (7.0)	<0.0001
Waist circumference (cm), %					
<80	18.1	0.9	14.3	67.9	
80–88	24.7	6.4	32.8	25.0	<0.0001
>88	57.2	92.7	52.9	7.1	
Waist-to-height ratio, mean (SD)	0.57 (0.08)	0.64 (0.07)	0.56 (0.07)	0.49 (0.04)	<0.0001
≥0.5, %	79.0	98.2	80.3	35.7	<0.0001
Fat mass (%), mean (SD)	35.2 (4.4)	36.8 (4.4)	35.2 (4.2)	32.1 (3.9)	<0.0001
>30, %	87.9	94.0	89.7	69.1	<0.0001
Place of residence, %					
village	27.9	30.3	30.7	10.7	
town (<20,000 inhabitants)	15.2	20.2	13.1	14.3	
town (20–100,000 inhabitants)	20.8	22.9	19.7	21.4	0.0085
city (>100,000 inhabitants)	36.2	26.6	36.5	53.6	
Education level, %					
primary	13.9	19.3	13.5	5.4	
secondary	57.7	59.6	57.8	53.6	0.0305
higher	28.4	21.1	28.7	41.1	
Economic situation, %					
below average	16.4	21.1	13.5	19.6	
average	70.4	68.8	73.0	62.5	0.2346
above average	13.2	10.1	13.5	17.9	
Situation of household, %					
we live poorly	0.2	0.0	0.0	1.8	
we live very thriftily	17.4	22.9	14.8	17.9	
we live thriftily	55.3	56.9	57.0	44.6	0.0684
we live well	24.9	19.3	25.8	32.1	
we live very well	2.2	0.9	2.5	3.6	
Socioeconomic status ^b^ (SES Index), mean (SD)	9.9 (2.1)	9.3 (2.1)	9.9 (2.1)	10.7 (2.2)	0.0004
Socioeconomic status ^b^, %					
low	40.6	54.1	38.5	23.2	
average	37.2	30.3	38.5	44.6	0.0025
high	22.2	15.6	23.0	32.1	
Physical activity at work ^c^, %					
low	53.8	72.5	48.4	41.1	
moderate	32.5	18.3	37.7	37.5	0.0001
high	13.7	9.2	13.9	21.4	
Physical activity in leisure time ^d^, %					
low	22.2	40.4	18.4	3.6	
moderate	64.3	58.7	68.0	58.9	<0.0001
high	13.4	0.9	13.5	37.5	
Overall physical activity ^e^, %					
low	52.6	81.7	47.1	19.6	
moderate	44.3	17.4	50.0	71.4	<0.0001
high	3.2	0.9	2.9	8.9	
Smokers ^f^, %	51.8	57.8	51.2	42.9	0.1830
Abuse of alcohol ^g^, %	4.2	7.3	3.3	1.8	0.1331
Age at menarche (years), %					
<12	11.7	14.7	12.3	3.6	
12–14.9	63.6	65.1	61.1	71.4	0.1881
≥15	24.7	20.2	26.6	25.0	
Age at menopause (years), %					
<40	2.3	3.2	1.5	4.1	
40–49.9	38.6	34.7	40.8	36.7	0.6448
≥50	59.1	62.1	57.8	59.2	
Number of full-term pregnancies, %					
0	11.7	7.3	11.9	19.6	
1–2	61.4	57.8	61.9	66.1	0.0250
≥3	26.9	34.9	26.2	14.3	
Breastfeeding time (months), %					
≤6	51.9	56.4	50.9	46.7	
7–12	24.7	21.8	26.2	24.4	
13–24	15.6	12.9	15.4	22.2	0.7795
>24	7.8	8.9	7.5	6.7	
Oral contraceptive use (ever), %	19.6	19.3	17.6	28.6	0.1757
Hormone-replacement therapy use (ever), %	16.9	11.0	18.0	23.2	0.1048
Family history of BC ^h^, %	18.8	20.2	18.0	19.6	0.9869
Vitamin/mineral supplement use ^i^, %	38.9	36.7	37.7	48.2	0.2991
Chronic disorders, %	57.0	60.6	55.3	57.1	0.6574

^a^ BMI was calculated using measured weight and height; ^b–e^ was described in Supplementary Table S2 [24,27,28]; ^f^ current or former-smokers; ^g^ at least 1 bottle (0.5 L) of beer or 2 glasses of wine (300 mL) or 2 drinks (300 mL) or 2 glasses of vodka (60 mL) consumption per day [5]; ^h^ in first- or second-degree relative; ^i^ self-declared use of vitamin and/or mineral supplements within the last 12 months; SD—standard deviation; %—sample percentage; *p*-value—level of significance assessed by Chi^2^ test (categorical variables) or Kruskal–Wallis test (continuous variables).

**Table 2:** The adherence to the QAd-WCRF/AICR recommendations and compliance with its single suggestions among breast cancer patients and controls (% or mean (SD).

Variable	Cancer-Control Sample	Cancer Sample	Control Sample	*p*-Value
Sample Size	409	179	230	
QAd-WCRF/AICR score (0–8 points), mean (SD)	4.1 (1.1)	3.8 (1.1)	4.3 (1.1)	<0.0001
QAd-WCRF/AICR score (0–8 points), %				
≤3	26.7	37.4	18.3	
4–5	59.7	53.6	64.3	<0.0001
6–8	13.7	8.9	17.4	
QAd-WCRF/AICR score (0–9 points) ^a^, mean (SD)	4.8 (1.1)	4.5 (1.1)	5.0 (1.1)	<0.0001
QAd-WCRF/AICR score (0–9 points) ^a^, %				
≤4	35.6	44.9	27.5	
5–6	54.2	47.3	60.1	0.0022
7–9	10.3	7.8	12.4	
BMI ^b^ (kg/m^2^), mean (SD)	27.9 (5.0)	28.3 (4.8)	27.6 (5.0)	0.1039
BMI ^b^ (kg/m^2^), %				
18.5–24.9	29.6	25.0	32.6	
25.0–29.9	38.4	40.4	37.8	0.2180
<18.5 or ≥30.0	32.0	34.6	29.6	
Waist circumference (cm), mean (SD)	92.0 (13.2)	94.0 (13.7)	90.4 (12.6)	0.0048
Waist circumference (cm), %				
<80	18.1	15.1	20.4	
80–88	24.7	21.8	27.0	0.0988
>88	57.2	63.1	52.6	
Overall physical activity ^c^, %				
high	3.2	1.6	4.3	
moderate	44.3	30.5	55.2	<0.0001
low	52.6	67.9	40.4	
Vegetables/fruits/whole grains/nuts/seeds/ legumes (frequency of consumption), mean (SD)	3.6 (1.5)	3.4 (1.5)	3.8 (1.5)	0.0017
Vegetables/fruits/whole grains/nuts/seeds/legumes (frequency of consumption), %				
>4 times/day	33.3	27.4	38.3	
2–4 times/day	53.6	54.2	53.0	0.0036
<2 times/day	13.1	18.4	8.7	
Highly processed food, including fast foods/sweets/instant soups (frequency of consumption), mean (SD)	0.6 (0.5)	0.6 (0.5)	0.5 (0.5)	0.0001
Highly processed food, including fast foods/sweets/instant soups (frequency of consumption), %				
≥1 time/day	21.0	26.8	16.1	
several times/week	59.0	61.1	57.4	0.0002
1–3 times/month or less	20.0	12.1	26.5	
Red and processed meat (frequency of consumption), mean (SD)	1.4 (0.8)	1.5 (0.8)	1.3 (0.8)	0.0071
Red and processed meat (frequency of consumption), %				
≥1 time/day	66.0	70.5	62.2	
several times/week	18.0	19.5	17.0	0.0102
1–3 times/month or less	16.0	10.0	20.9	
Sugar-sweetened and energy drinks (frequency of consumption), mean (SD)	0.1 (0.2)	0.1 (0.2)	0.0 (0.1)	0.4865
Sugar-sweetened and energy drinks (frequency of consumption), %				
≥1 time/day	1.4	2.6	0.4	
several times/week	4.5	5.3	3.9	0.1305
1–3 times/month or less	94.0	92.1	95.7	
Alcohol (frequency of consumption), mean (SD)	0.1 (0.1)	0.1 (0.1)	0.1 (0.1)	0.1193
Alcohol (frequency of consumption), %				
≥1 time/day	0.2	0.5	0.0	
<1 time/day	59.3	46.8	69.6	<0.0001
abstinence	40.5	52.6	30.4	
For mothers: breastfeeding if you can, %				
>6 months	47.8	44.6	50.8	
≤6 months	52.2	55.4	49.2	0.2340

^a^ including optional component for mothers: breastfeeding if you can (*n* = 360 respondents, including 167 breast cancer cases and 193 controls); ^b^ BMI was calculated using measured weight and height; ^c^ after combining data based on declared physical activity at work and physical activity during leisure time [28]; the frequency of consumption of various food groups was expressed as summarised as times/day, after assigning the values for categories of frequencies as follows: ‘never or almost never’ = 0; ‘once a month or less’ = 0.025; ‘several times a month’ = 0.1; ‘several times a week’ = 0.571; ‘daily’ = 1; ‘several times a day’ = 2; SD—standard deviation; %—sample percentage; *p*-value—level of significance assessed by Chi^2^ test (categorical variables) or Kruskal–Wallis test (continuous variables).

**Table 3:** Odds ratios (ORs (95% CI) of breast cancer occurrence resulting from adherence to the QAd-WCRF/AICR recommendations for peri- and postmenopausal women.

Variable	Adherence	Sample Size	Control	Breast Cancer					
			OR	OR_crude_	95% CI	*p*-Value	OR_adj_	95% CI	*p*-Value
QAd-WCRF/AICR Score (0–8 points)	Three-level division								
	minimal (≤3; ref.)	109	Ref.	Ref.			Ref.		
	moderate (4–5)	244	1.00	0.41	0.26; 0.65	0.0001	0.46	0.28; 0.76	0.0024
	maximal (6–8)	56	1.00	0.25	0.12; 0.51	<0.0001	0.28	0.13; 0.63	0.0018
	Two-level division								
	lower (0–4; ref.)	237	Ref.	Ref.			Ref.		
	higher (5–8)	172	1.00	0.43	0.28; 0.65	<0.0001	0.49	0.31; 0.76	0.0015
	score (1-point increase)		1.00	0.63	0.52; 0.76	<0.0001	0.69	0.56; 0.86	0.0007
QAd-WCRF/AICR Score (0–9 points ^a^)	Three-level division								
	minimal (≤4; ref.)	128	Ref.	Ref.			Ref.		
	moderate (5–6)	195	1.00	0.32	0.12; 0.84	0.0199	0.35	0.13; 0.98	0.0445
	maximal (7–9)	37	1.00	0.15	0.05; 0.40	0.0002	0.17	0.06; 0.54	0.0023
	Two-level division								
	lower (0–5; ref.)	247	Ref.	Ref.			Ref.		
	higher (6–9)	113	1.00	0.41	0.26; 0.66	0.0002	0.45	0.27; 0.75	0.0020
	score (1-point increase)		1.00	0.66	0.54; 0.80	<0.0001	0.71	0.57; 0.88	0.0019

^a^ including optional component for mothers: breastfeeding if you can (*n* = 360 respondents, including 167 breast cancer cases and 193 controls); ref.—referent; the reference categories included the control sample and the minimal or lower adherence to the WCRF/AICR score; OR_crude_—crude model; OR_adj_—age (years), socioeconomic status (low, average, high), smoking status (non-smoker, smoker), age at menarche (<12, 12–14.9, ≥15 years), menopausal status (peri-, postmenopausal), number of full-term pregnancies (0, 1–2, ≥3), oral contraceptive use (no, yes), hormone-replacement therapy use (no, yes), family history of breast cancer in first- or second-degree relative (no, I don’t know, yes), chronic diseases (no, yes), vitamin/mineral supplements use (no, yes) and an indication of the breast cancer subtype (triple negative, ER-, PR-, HER2+ subtype, luminal A, luminal B) adjusted model; 95% CI—95% confidence interval; *p*-value—the level of significance was assessed using the Wald test.

**Table 4:** Odds ratios (ORs (95% CI) of breast cancer occurrence by compliance with single QAd-WCRF/AICR recommendations among peri- and postmenopausal women.

QAd-WCRF/AICR Recommendations	Categories	Sample Size	Control	Breast Cancer								
			OR	OR_crude_	95% CI	*p*-Value	OR_adj_	95% CI	*p*-Value	OR_m-adj_	95% CI	*p*-Value
BMI (kg/m^2^)	<18.5 or ≥30.0 (ref.)	130	Ref.	Ref.			Ref.			Ref.		
	25.0–29.9	160	1.00	0.91	0.57; 1.47	0.7076	0.92	0.54; 1.56	0.7612	1.15	0.55; 2.41	0.7141
	18.5–24.9	119	1.00	0.66	0.40; 1.08	0.0970	0.65	0.36; 1.16	0.1441	0.86	0.32; 2.29	0.7645
	1-unit increase		1.00	1.03	0.99; 1.07	0.1526	1.01	0.97; 1.06	0.5395	0.98	0.92; 1.04	0.5337
WC (cm)	>88 (ref.)	234	Ref.	Ref.			Ref.			Ref.		
	80–88	101	1.00	0.67	0.42; 1.09	0.1033	0.77	0.46; 1.29	0.3270	0.96	0.50; 1.83	0.8948
	<80	74	1.00	0.62	0.36; 1.06	0.0769	0.71	0.39; 1.30	0.2633	0.76	0.31; 1.87	0.5536
	1-unit increase		1.00	1.02	1.01; 1.04	0.0056	1.02	1.00; 1.04	0.0378	1.04	1.01; 1.07	0.0206
Overall physical activity	low (ref.)	216	Ref.	Ref.			Ref.			Ref.		
	moderate or high	193	1.00	0.32	0.21; 0.48	<0.0001	0.35	0.22; 0.55	<0.0001	0.33	0.20; 0.56	<0.0001
Vegetables/fruits/whole grains/nuts/seeds/legumes (frequency of consumption)	<2 times/day (ref.)	54	Ref.	Ref.			Ref.			Ref.		
	2–4 times/day	219	1.00	0.48	0.26; 0.90	0.0189	0.55	0.29; 1.04	0.0643	0.59	0.28; 1.25	0.1645
	>4 times/day	136	1.00	0.34	0.18; 0.65	0.0010	0.41	0.20; 0.86	0.0172	0.36	0.15; 0.86	0.0208
	1-unit increase		1.00	0.82	0.72; 0.94	0.0032	0.88	0.76; 1.01	0.0707	0.91	0.77; 1.07	0.2387
Highly processed food, including fast foods/sweets/instant soups (frequency of consumption)	≥1 time/day (ref.)	86	Ref.	Ref.			Ref.			Ref.		
	several times/week	241	1.00	0.64	0.39; 1.04	0.0726	0.57	0.33; 0.97	0.0388	0.53	0.28; 0.99	0.0440
	1–3 times/month or less	82	1.00	0.27	0.14; 0.52	<0.0001	0.31	0.15; 0.64	0.0013	0.36	0.15; 0.83	0.0165
	1-unit increase		1.00	1.77	1.18; 2.66	0.0053	1.64	1.05; 2.57	0.0306	1.63	0.97; 2.73	0.0624
Red and processed meat (frequency of consumption)	≥1 time/day (ref.)	268	Ref.	Ref.			Ref.			Ref.		
	several times/week	75	1.00	1.01	0.56; 1.82	0.9670	0.91	0.52; 1.57	0.7266	0.92	0.49; 1.71	0.7873
	1–3 times/month or less	66	1.00	0.42	0.24; 0.76	0.0037	0.48	0.25; 0.91	0.0235	0.57	0.26; 1.23	0.1490
	1-unit increase		1.00	1.42	1.11; 1.82	0.0056	1.31	1.01; 1.71	0.0449	1.16	0.84; 1.60	0.3783
Sugar-sweetened and energy drinks (frequency of consumption) ^a^	≥1 time/day or several times/week (ref.)	25	Ref.	Ref.			Ref.			Ref.		
	1–3 times/month or less	384	1.00	0.53	0.23; 1.21	0.1315	0.54	0.22; 1.34	0.1829	0.71	0.25; 2.06	0.5311
	1-unit increase		1.00	2.76	0.86; 8.90	0.0886	2.29	0.62; 8.41	0.2114	1.62	0.37; 7.20	0.5234
Alcohol (frequency of consumption) ^b^	<1 time/day (ref.)	242	Ref.	Ref.			Ref.			Ref.		
	abstinence	166	1.00	2.57	1.72; 3.84	<0.0001	2.50	1.60; 3.91	<0.0001	2.98	1.78; 4.98	<0.0001
	1-unit increase		1.00	0.28	0.06; 1.43	0.1250	0.41	0.07; 2.36	0.3171	0.19	0.02; 2.11	0.1760
For mothers: breastfeeding if you can	≤6 months (ref.)	188	Ref.	Ref.			Ref.			Ref.		
	>6 months	172	1.00	0.78	0.52; 1.18	0.2341	0.81	0.52; 1.28	0.3681	0.92	0.57; 1.50	0.7451

ref.—referent, the reference categories were the control sample and lack of compliance with the QAd-WCRF/AICR single recommendations; OR_crude_—crude model; OR_adj_—age (years), socioeconomic status (low, average, high), smoking status (non-smoker, smoker), age at menarche (<12, 12–14.9, ≥15 years), menopausal status (peri-, postmenopausal), number of full-term pregnancies (0, 1–2, ≥3), oral contraceptive use (no, yes), hormone-replacement therapy use (no, yes), family history of breast cancer in first- or second-degree relative (no, I don’t know, yes), chronic diseases (no, yes), vitamin/mineral supplements use (no, yes) and indication of breast cancer subtype (triple negative, ER-, PR-, HER2+ subtype, luminal A, luminal B) adjusted model; OR_m-adj_—multi-variable adjusted model, including the set of confounders of the OR_adj_ model and the remaining components of the WCRF/AICR score (continuous BMI, WC, and food consumption, and original categories for physical activity and breastfeeding); ^a^ due to the small sample size of the reference category, the lowest category and the intermediate category were merged together and considered as the reference category; ^b^ due to the small sample size, the lowest category was excluded, and the intermediate category was considered as the reference; 95% CI—95% confidence interval; *p*-value—the level of significance was assessed using the Wald test.

## Data Availability

The raw data supporting the conclusions of this article will be made available by the authors on request.

## Supplementary Materials

The following supporting information can be downloaded at: https://www.mdpi.com/2072-6694/16/2/468/s1, Table S1: The original 2018 WCRF/AICR recommendations and the qualitative-adapted 2018 WCRF/AICR recommendations (QAd-WCRF/AICR Score); Table S2: Socioeconomic status and overall physical activity categories in the case–control study regarding associations of the adherence to the qualitative-adapted 2018 WCRF/AICR recommendations (QAd-WCRF/AICR Score) and breast cancer risk among peri- and postmenopausal women; Table S3: Characteristics of adherence to the qualitative-adapted 2018 WCRF/AICR recommendations (QAd-WCRF/AICR Score), according to its single recommendations (% or mean (SD). References [24,25,26,27,28] are cited in the supplementary materials.
