# Supplementary material for: Compliance with the WCRF/AICR Recommendations in Qualitative Adaptation and the Occurrence of Breast Cancer: A Case-Control Study
Source: Cancers (Basel). 2024 Jan 22;16(2):468. doi: 10.3390/cancers16020468 (PMC11154306; doi:10.3390/cancers16020468)
Supplement: Supplementary file 1 [file cancers-16-00468-s001.zip › cancers-2808096-supplementary.doc]

**Table S1**. The original 2018 WCRF/AICR Score and the qualitative-adapted 2018 WCRF/AICR Score (QAd-WCRF/AICR Score)

| No. of recommendations | 2018 WCRF/AICR recommendations | 2018 WCRF/AICR Score [1,2] | Points | QAd-WCRF/AICR Score | Points |
| --- | --- | --- | --- | --- | --- |
| 1. | Be a healthy weight | BMI (kg/m^2^) |  | BMI (kg/m^2^) |  |
|  |  | 18.5-24.9 | 0.5 | 18.5-24.9 | 1 |
|  |  | 25.0-29.9 | 0.25 | 25.0-29.9 | 0.5 |
|  |  | <18.5 or ≥30.0 | 0 | <18.5 or ≥30.0 | 0 |
|  |  | Waist circumference (cm) |  | Waist circumference (cm) |  |
|  |  | <80 | 0.5 | <80 | 1 |
|  |  | 80- <88 | 0.25 | 80-88 | 0.5 |
|  |  | ≥88 | 0 | >88 | 0 |
| 2. | Be physically active | Total moderate-vigorous physical activity (min/week) |  | Overall physical activity^&^ |  |
|  |  | ≥150 | 1 | high | 1 |
|  |  | 75-<150 | 0.5 | moderate | 0.5 |
|  |  | <75 | 0 | low | 0 |
| 3. | Eat a diet rich in whole grains, vegetables, fruits and beans | Fruits and vegetables (g/day) |  | Vegetables/fruits/wholegrains/nuts/seeds/legumes  (frequency of consumption) |  |
|  |  | ≥400 | 0.5 | >4 times/day | 1 |
|  |  | 200-<400 | 0.25 | 2-4 times/day | 0.5 |
|  |  | <200 | 0 | <2 times/day | 0 |
|  |  | Total fiber (g/day) |  |  |  |
|  |  | ≥30 | 0.5 |  |  |
|  |  | 15-<30 | 0.25 |  |  |
|  |  | <15 | 0 |  |  |
| 4. | Limit consumption of “fast foods” and other processed foods high in fat, starches or sugars | Percent of total kcal from ultra-processed foods: |  | Highly processed food including fast foods/sweets/instant soups  (frequency of consumption) |  |
|  |  | Tertile 1 | 1 | ≤ several times/month | 1 |
|  |  | Tertile 2 | 0.5 | several times/week | 0.5 |
|  |  | Tertile 3 | 0 | ≥1 time/day | 0 |
| 5. | Limit consumption of red and processed meat | Total red meat (g/week) and processed meat (g/week): |  | Red and processed meat  (frequency of consumption) |  |
|  |  | Red meat <500 and processed meat <21 | 1 | ≤ several times/month | 1 |
|  |  | Red meat <500 and processed meat 21-<100 | 0.5 | several times/week | 0.5 |
|  |  | Red meat >500 or processed meat ≥100 | 0 | ≥1 time/day | 0 |
| 6. | Limit consumption of sugar-sweetened drinks | Total sugar-sweetened drinks (g/day) |  | Sugar-sweetened and energy drinks (frequency of consumption) |  |
|  |  | 0 | 1 | ≤ several times/month | 1 |
|  |  | 0-≤250 | 0.5 | several times/week | 0.5 |
|  |  | >250 | 0 | ≥1 time/day | 0 |
| 7. | Limit alcohol consumption | Total ethanol (g/day) |  | Alcohol (frequency of consumption) |  |
|  |  | 0 | 1 | abstinence | 1 |
|  |  | >0 and ≤14 (1 drink) | 0.5 | <1 time/day | 0.5 |
|  |  | >14 (1 drink) | 0 | ≥1 time/day | 0 |
| 8. | (Optional) For mothers: breastfeed your baby, if you can | Exclusive breastfeeding over lifetime for a total of: |  | Breastfeeding (the longest period of time) |  |
|  |  | ≥6 months | 1 | >6 months | 1 |
|  |  | >0- <6 months | 0.5 | ≤6 months | 0.5 |
|  |  | never | 0 | never | 0 |
| **Total Score Range**: | | | **0–7**  or **0–8** |  | **0–8**  or **0–9** |

AICR – American Institute for Cancer Research; WCRF – World Cancer Research Fund; BMI – body mass index; ^&^after combining data based on physical activity at work and physical activity in leisure time (Supplementary Table S2);

[1] Shams-White MM, Brockton NT, Mitrou P, Romaguera D, Brown S, Bender A, Kahle L, Reedy J. [Operationalizing the 2018 World Cancer Research Fund/American Institute for Cancer Research (WCRF/AICR) Cancer Prevention Recommendations: A Standardized Scoring System](https://pubmed.ncbi.nlm.nih.gov/31336836/). Nutrients. 2019;11(7).

[2] Shams-White MM, Romaguera D, Mitrou P, Reedy J, Bender A, Brockton NT. [Further Guidance in Implementing the Standardized 2018 World Cancer Research Fund/American Institute for Cancer Research (WCRF/AICR) Score](https://pubmed.ncbi.nlm.nih.gov/32152215/). Cancer Epidemiol. Biomarkers Prev. 2020;29(5):889-94.

**Table S2**. Socioeconomic status and overall physical activity categories in the case-control study regarding associations of the adherence to the qualitative-adapted 2018 WCRF/AICR Score (QAd-WCRF/AICR Score) and breast cancer occurrence among peri- and postmenopausal women [3-5]

| **Variable** | **Categories** | |
| --- | --- | --- |
| **Socioeconomic status (SES)**  after combining data based on SES’ factors: | low; average; high | |
| place of residence | village | |
|  | town <20,000 inhabitants | |
|  | town 20,000-100,000 inhabitants | |
|  | city >100,000 inhabitants | |
| educational level | primary | |
|  | secondary | |
|  | higher | |
| economic situation  (self-declared) | below average | |
|  | average | |
|  | above average | |
| situation of household (self-declared) | we live poorly – I don’t have enough resources even for basic needs (food/clothing/housing fees) | |
|  | we live very thriftily – I have enough resources only for basic needs (food/clothing/housing fees) | |
|  | we live thriftily – so I have enough resources for everything | |
|  | we live well – I have enough resources for everything, but I don’t put off savings | |
|  | we live very well – I have enough resources for everything and I put off savings | |
| **Overall physical activity^&^** | low; moderate; high | |
| physical activity at work | low | more than 70% of working time spent sedentary or retired |
|  | moderate | approx. 50% of working time spent sedentary and 50% of working time spent in an active manner |
|  | high | approx. 70% of working time spent in an active manner or physical work related to great exertion |
| physical activity in leisure time | low | sedentary for most of the time, watching TV, reading books, walking 1-2 hours per week |
|  | moderate | walking, bike riding, gymnastics, gardening, light physical activity performed 2-3 hours per week |
|  | high | bike riding, jogging, gardening, sport activities involving physical exertion performed more than 3 hours weekly |

**^&^** after combining data based on physical activity at work and physical activity in leisure time

[3] Krusinska, B.; Wadolowska, L.; Slowinska, M.A.; Biernacki, M.; Drozdowski, M.; Chadzynski, T. Associations of dietary patterns and metabolic-hormone profiles with breast cancer risk: A case-control study. Nutrients 2018, 10, 2013, doi:10.3390/nu10122013.

[4] Jarosz, M.; Taraszewska, A. Nadwaga i otyłosc oraz wybrane elementy stylu zycia jako czynniki ryzyka GERD. (Overweight and obesity and selected lifestyle elements as risk factors for GERD). Postepy Nauk Medycznych 2011, 9, 749–759. (In Polish)

[5] Wadolowska L., Stasiewicz B. The manual for developing nutritional data from the KomPAN® questionnaire. Chapter 3. (in:) KomPAN® Dietary Habits and Nutrition Beliefs Questionnaire and the manual for developing nutritional data. Ed. Gawecki J. The Committee of Human Nutrition, Polish Academy of Sciences, Olsztyn, 2020, 35-56. Available at: http://www.knozc.pan.pl/

**Table S3.** Characteristics of the adherence to the qualitative-adapted 2018 WCRF/AICR Score (QAd-WCRF/AICR Score) by its single recommendations (% or mean (SD)

| **Variable** | **Adherence to the QAd-WCRF/AICR Score**  **(0-8 points)** | | | ***p*-Value** |
| --- | --- | --- | --- | --- |
|  | **Minimal**  **(≤3)** | **Moderate**  **(4-5)** | **Maximal**  **(6-8)** |  |
| Sample Size | 109 | 244 | 56 |  |
| QAd-WCRF/AICR Score (0-8points)^#^ | 2.8 (0.3) | 4.2 (0.5) | 6.0 (0.6) | <0.0001 |
| BMI^a^ (kg/m^2^)*^#^* | 31.6 (4.6) | 27.4 (4.5) | 23.1 (2.1) | <0.0001 |
| 18.5-24.9 | 1.8 | 29.9 | 82.1 |  |
| 25.0-29.9 | 33.9 | 45.5 | 16.1 | <0.0001 |
| <18.5 or ≥30.0 | 64.2 | 24.6 | 1.8 |  |
| Waist circumference (cm)^#^ | 102.6 (11.5) | 90.3 (11.2) | 78.4 (7.0) | <0.0001 |
| <80 | 0.9 | 14.3 | 67.9 |  |
| 80-88 | 6.4 | 32.8 | 25.0 | <0.0001 |
| >88 | 92.7 | 52.9 | 7.1 |  |
| Overall physical activity^b^ |  |  |  |  |
| high | 0.9 | 2.9 | 8.9 |  |
| moderate | 17.4 | 50.0 | 71.4 | <0.0001 |
| low | 81.7 | 47.1 | 19.6 |  |
| Eat vegetables/fruits/wholegrains/nuts/seeds/  legumes (times/day)^#^ | 2.9 (1.1) | 3.8 (1.5) | 4.6 (1.7) | <0.0001 |
| >4 times/day | 13.8 | 36.9 | 60.7 |  |
| 2-4 times/day | 57.8 | 54.1 | 39.3 | <0.0001 |
| <2 times/day | 28.4 | 9.0 | 0.0 |  |
| Limit highly processed food including fast foods/sweets/instant soups (times/day)^#^ | 0.73 (0.5) | 0.53 (0.5) | 0.32 (0.3) | <0.0001 |
| ≥1 time/day | 35.8 | 17.6 | 5.4 |  |
| several times/week | 60.6 | 59.4 | 55.4 | <0.0001 |
| 1-3 times/month or less | 3.7 | 23.0 | 39.3 |  |
| Limit red and processed meat (times/day)^#^ | 1.7 (0.7) | 1.3 (0.7) | 0.7 (0.7) | <0.0001 |
| ≥1 time/day | 86.2 | 64.3 | 32.1 |  |
| several times/week | 13.8 | 19.7 | 19.6 | <0.0001 |
| 1-3 times/month or less | 0.0 | 16.0 | 48.2 |  |
| Limit sugary and energy drinks (times/day)^#^ | 0.1 (0.2) | 0.0 (0.1) | 0.0 (0.0) | 0.0002 |
| ≥1 time/day | 2.8 | 1.2 | 0.0 |  |
| several times/week | 10.1 | 2.5 | 0.0 | 0.0027 |
| 1-3 times/month or less | 87.2 | 96.3 | 100.0 |  |
| Limit alcohol consumption (times/day)^#^ | 0.1 (0.1) | 0.1 (0.1) | 0.1 (0.1) | 0.3925 |
| ≥1 time/day | 0.0 | 0.4 | 0.0 |  |
| <1 time/day | 64.2 | 59.8 | 44.6 | 0.1462 |
| abstinence | 35.8 | 39.8 | 55.4 |  |
| For mothers: breastfeeding if you can |  |  |  |  |
| >6 months | 43.6 | 49.1 | 53.3 |  |
| ≤6 months | 56.4 | 50.9 | 46.7 | 0.4952 |

^a^BMI was calculated using measured weight and height; ^b^after combining data based on declared physical activity at work and physical activity in leisure time [4,5]; the frequency consumption of food groups was expressed as summarized times/day after assigning the values for categories of frequencies as follows: ‘never or almost never’=0; ‘once a month or less’=0.025; ‘several times a month’=0.1; ‘several times a week’=0.571; ‘daily’=1; ‘several times a day’=2; % – sample percentage; ^#^mean (SD); p-value – level of significance assessed by chi^2^ test (categorical variables) or Kruskal-Wallis’ test (continuous variables).
